# Awns reduce grain number to increase grain size and harvestable yield in irrigated and rainfed spring wheat

**DOI:** 10.1093/jxb/erw081

**Published:** 2016-03-14

**Authors:** G. J. Rebetzke, D. G. Bonnett, M. P. Reynolds

**Affiliations:** ^1^CSIRO Agriculture, PO Box 1600, Canberra, ACT 2601, Australia; ^2^Bayer Crop Science, 6693 90th St Sabin, MN 56580USA; ^3^CIMMYT Int. Apdo. Postal 6–641, 06600 México, DF, Mexico

**Keywords:** Breeding, canopy temperature, drought, germplasm, harvest index, heritability, photosynthesis, screenings, test weight.

## Abstract

Genotypic variation in ear morphology is linked to differences in photosynthetic potential to influence grain yield in winter cereals. Awns contribute to photosynthesis, particularly under water-limited conditions when canopy assimilation is restricted. We assessed performance of up to 45 backcross-derived, awned–awnletted NILs representing four diverse genetic backgrounds in 25 irrigated or rainfed, and droughted environments in Australia and Mexico. Mean environment grain yields were wide-ranging (1.38–7.93 t ha^−1^) with vegetative and maturity biomass, plant height, anthesis date, spike number, and harvest index all similar (*P* >0.05) for awned and awnletted NILs. Overall, grain yields of awned–awnletted sister-NILs were equivalent, irrespective of yield potential and genetic background. Awnletted wheats produced significantly more grains per unit area (+4%) and per spike (+5%) reflecting more fertile spikelets and grains in tertiary florets. Increases in grain number were compensated for by significant reductions in grain size (–5%) and increased frequency (+0.8%) of small, shrivelled grains (‘screenings’) to reduce seed-lot quality of awnletted NILs. Post-anthesis canopies of awnletted NILs were marginally warmer over all environments (+0.27 °C) but were not different and were sometimes cooler than awned NILs at cooler air temperatures. Awns develop early and represented up to 40% of total spikelet biomass prior to ear emergence. We hypothesize that the allocation of assimilate to large and rapidly developing awns decreases spikelet number and floret fertility to reduce grain number, particularly in distal florets. Individual grain size is increased to reduce screenings and to increase test weight and milling quality, particularly in droughted environments. Despite the average reduction in grain size, awnless lines could be identified that combined higher grain yield with larger grain size, increased grain protein concentration, and reduced screenings.

## Abstract (precis)

Awns were linked to larger grain size, improved seedlot quality, and yield in less favourable environments but trade-offs in grain number reduced much of their benefit in more favourable environments.

## Introduction

A number of traits have been identified with potential to improve the water productivity of winter cereals. These traits span the continuum from seed germination and early growth to floral development and flowering and then carbon accumulation and remobilization during grain-filling (Araus *et al.*, 2002; Richards *et al.*, 2002; Reynolds and Tuberosa, 2008). Perhaps the greatest focus to date has been on factors contributing to changes in shoot growth and the acquisition and efficient use of water (Condon *et al.*, 2004), the accumulation and remobilization of stem carbohydrates (Rebetzke *et al.*, 2008), phasic development (Borràs-Gelonch *et al.*, 2012; Cane *et al.*, 2013), and, more recently, altered root architecture (Wasson *et al.*, 2012; Pinto and Reynolds, 2015).

As the extent, nature, and value of genotypic variation in shoot growth becomes better understood for deployment and selection in breeding programmes, new opportunities are being explored toward improved cereal performance in water-limited environments (Maydup *et al.*, 2014). Interest is growing in the role of the ear to support carbon assimilation in the upper canopy (Sanchez-Bragado *et al.*, 2014) or to provide additional capacity after leaf senescence arising through drought, hot winds, and/or leaf disease (Tambussi *et al.*, 2007). The potential contribution of the ear could be large with awned-spikes intercepting between 18% and 45% of incident radiation during grain-filling depending on the genotype (Lopez-Castañeda *et al.*, 2014).

Awns, threadlike extensions of the lemma, increase the surface area of the ear in bread wheat by up to 50% (Blum, 1985) and by up to 60% in longer-awned, durum wheat (Blum, 1985; Motzo and Giunta, 2002). In carefully managed field experiments contrasting awned and awnless NILs, Evans *et al.* (1972) reported that awns doubled the net photosynthetic rate of spikes during grain-filling under irrigated conditions whereas, under drought, the proportion of spike-contributed photosynthesis increased from between 13–24% in awnless ears, and between 34–43% in awned ears. Similarly, awns contributed up to 50% of the total spike carbon exchange rate in bread and durum wheats in stress-free conditions (Blum, 1985) and, more recently, up to 42% of grain yield in longer-awned bread wheat varieties (Maydup *et al.*, 2010). The potential need for additional ear photosynthesis can be large, with numerous environmental conditions inhibiting canopy photosynthesis including water limitation, high air temperatures, leaf diseases, and other causes of premature leaf senescence (Martin *et al.*, 1993).

The glumes and awns may then represent significant (and sometimes the only) photosynthetic tissue with the potential to fix atmospheric carbon through grain-filling. The unique anatomical structure of awns is speculated to contribute to greater water-use efficiency (WUE) than the flag leaf (Blum, 1985; Bort *et al.*, 1994) suggesting more efficient assimilation per unit of water transpired which is important in water-limited crop production. These anatomical differences are also believed to contribute to greater high temperature tolerance of awn rather than leaf tissue (Blum, 1986) while direct vascular linkage between the awns and the lemma should permit direct carbon movement to the developing grain (Evans *et al.*, 1972; Li *et al.*, 2010*a*).

Long awns are considered to be an important component trait of the high-yielding wheat ideotype, particularly for wheat grown under water-limited conditions (Reynolds and Tuberosa, 2008). Greater WUE, thermo-tolerance, and intrinsically higher photosynthetic potential of awns should translate to significantly greater yield potential for awned wheat varieties. In turn, awns should be ubiquitous to all bread wheat varieties. However, awnless wheats only predominate in some global production areas. Studies contrasting related awned and awnless lines have demonstrated a grain yield advantage for awned wheats of up to 16% (Olugbemi *et al.*, 1976; Martin *et al.*, 1993; Motzo and Giunta, 2002) and are supported by awn removal studies of awned varieties (Chhabra and Sethi, 1989). Teare and Petersen (1971) reported a stronger association for grain yield and awn surface area (*r*_p_=0.72) than for grain yield and flag leaf area (*r*_p_=0.49) for 30 field-grown wheat varieties. By contrast, other studies show that awns were associated with a yield reduction (McKenzie, 1972; Teich, 1982; Knott, 1986; Hosseini *et al.*, 2012), and/or that yield benefits were not repeatable across genetic backgrounds (Patterson *et al.*, 1962; Martin *et al.*, 1993; Weyhrich *et al.*, 1994). An anecdotal finding is that awned wheats tend to perform better in drier, warmer environments and awnless wheats in cooler, temperate environments (Teich, 1982).

Many of the reported benefits of awns have been from studies based on unrelated varieties or only a few near-isogenic lines with assessment restricted to glasshouse or a few outdoor environments. The aims of this study were to assess the influence of awns on grain yield and agronomic performance in large field plots for awned–awnletted NILs representing a range of diverse spring wheat genetic backgrounds. These NILs were assessed across a wide range of potential yield environments with the secondary aim to investigate whether any benefit of awns was specific to drier, warmer environments.

## Materials and methods

### Development of near-isogenic pairs

Four populations were developed containing multiple NIL pairs varying for the presence and absence of full awns. Crosses were initially generated between an awnletted donor ‘HM14bS’ (Halberd*2/Mara) and four genetically-diverse commercial spring wheat varieties Frame, Janz, Silverstar, and Westonia. The F_1_ seeds were harvested, sown, and then backcrossed to each of the respective commercial parents. Resulting BC_1_F_1_ seeds were sown and allowed to self-pollinate to produce *c.* 400 BC_1_F_2_ progeny per background. The F_2_ progeny were harvested and underwent single-seed descent without selection for three generations to produce 400 BC_1_F_4:5_ individuals. Awnletted plants were harvested and then threshed separately before sowing into F_5:6_ rows. Four to six awned and awnletted heads were harvested from segregating rows, threshed, and sown into F_6:7_ rows for progeny testing. Rows uniform for the presence or absence of awns were then harvested to develop F_6_-derived, F_7:8_ pairs of NILs. Seeds from these plants were individually threshed before sowing into rows in the winter of 2001 for awn, flowering date, and plant height assessment. Up to 12 NILs were sampled in each background to produce 45 BC_1_F_5_-derived, F_7:8_ NIL pairs. The resulting co-ancestry for any random allele across a NIL pair was estimated at 99.2% and, since the pairs themselves were related through the BC_1_F_2_, co-ancestry between NILs within a background was 75%.

### Experiment management

Four separate sets of experiments representing a total of 25 environments (23 in Australia and two in Mexico) were conducted, depending on the germplasm and environments sampled. Three sets of experiments (irrigated and rainfed, and Managed Environments) representing different seasons and numbers of genotypes were conducted at various locations in the wheat belts of southern and northern Australia and a fourth set containing a droughted and irrigated treatment at Obregon in north-west Mexico.

In Australia, sowing occurred in May and experiments harvested in December. In total, 23 environments were sampled representing multiple years and sites. In the first three sets of experiments, from 2002 to 2004 and again in 2011 and 2012, all 45 NILs were sown at sites including Ginninderra Experiment Station (GES) in ACT, Griffith, Gundibindyal, Narrabri, and Yanco in NSW, and Gatton in QLD. In a third set of experiments, 16 of the 45 lines (four NIL pairs×four genetic backgrounds) were sown in 2012 and 2013 at Merredin (Western Australia), Narrabri (NSW), and Yanco (NSW) as part of the national Managed Environment Facilities (MEFs; Rebetzke *et al.*, 2013*a*). At each MEF, the response of the individual NILs was ascertained in both a well-managed rainfed and an irrigated experiment.

Soil types were a red-brown earth of slightly acid to neutral soil pH except at Gatton and Narrabri where the soils were black vertisols. Crops were commonly sown after canola (*Brassica napus* L.) or alfalfa (*Medicago sativa* L.) break-crops to minimize the incidence of root disease and managed with adequate nutrition and pesticides to control weeds and leaf diseases. Experiments were multiple-augmented designs with the parents (Frame, Janz, Silverstar, and Westonia) replicated up to eight times throughout each experiment. In all studies, entries were sown at an optimal 3–5cm sowing depth into 6-m long, 0.17-m spaced, 5-row plots at a seeding rate of *c.* 200 seeds m^–2^. Nutrients were supplied at sowing as Starter 15^®^ (14% N:12.7% P:11% S) applied at 103kg ha^−1^. Additional nitrogen was applied as needed to meet crop demand and to ensure that grain protein was achieved at industry standards of 11.5% and above (data not shown). Plots at Gundibindyal were wholly reliant on pre- and growing-season rainfall whereas supplemental irrigation of 25-40mm was supplied up to flowering and during grain-filling at Gatton QLD, GES, Griffith, and Yanco. Irrigation at each MEF was scheduled to provide only enough soil water through the season to produce a 10–20% yield benefit in the irrigated compared with the rainfed treatments (Rebetzke *et al.*, 2013*a*).

In Mexico, two co-located experiments were conducted in 2007 under an irrigated and drought watering regime on a black vertisol at CIMMYT in Obregon, NW Mexico. These experiments were conducted on beds containing two rows spaced 20cm apart, and beds spaced 80cm apart centre to centre. Nutrients and pesticides were supplied when necessary. Both irrigated and droughted regimes were sown on full soil-water profiles with 30-40mm irrigation supplied every 3–4 weeks on the irrigated regime. All awned or awnletted NILs were grown, with the exception of the Silverstar genetic background, and replicated twice in a Randomized Complete Block Design.

### Field trait phenotyping

Early ground cover (Botwright *et al.*, 2002) and Normalized Difference Vegetation Index (NDVI) scores were undertaken at the different MEF at five growth stages from soon after emergence (Z12) to late-tillering (Z45). Groundcover was assessed using digital images taken with a Nikon® digital camera before converting to percentage ground cover estimates using the vegetation-cover prediction software ‘CanopyCover’ (Li *et al.*, 2010*a*). Parameters in the prediction model were set to minimize the background signal from the soil. The NDVI measurements were made using a GreenSeeker®. For all environments, phenological development near anthesis was recorded using a Zadoks score (Zadoks *et al.*, 1974). Canopy temperatures (CT) were undertaken at full ear emergence but before flowering (Z55–60) under irrigation at GES and Gatton (in 2004), Yanco (2011), under both drought and irrigation in Mexico, and pre- and post-flowering under drought and irrigation in the MEF. Measurements were made between 10.00h and 12.00h on still, cloud-free days. A single, separate CT assessment was also undertaken one night between 20.00h and 21.00h at GES in 2004. A Mikron® infrared thermometer was used to record CT after Rebetzke *et al.* (2013*b*). At anthesis, biomass cuts were undertaken in 11 environments before drying at 72 °C for 3 d. Dried samples were weighed and water-soluble carbohydrate analysis undertaken on bulk samples after Rebetzke *et al.* (2008). At physiological maturity, scoring was undertaken of plot lodging (where 1=vertical crop to 9=prostrate to the soil surface), and plant height measured as the distance from the soil surface to the top of the ear (awns excluded) of the tallest culms for each plot. At harvest maturity, *c.* 120 culms were hand-cut at ground level using a 40-cm quadrat oriented across four bordered rows. Harvested samples were air-dried at 35 °C for 3 d and weighed before and after careful threshing to retain all grains and harvest index was calculated as the ratio of grain weight to total above-ground biomass. Spike number was also counted for this harvest sample. The percentage of small or shrivelled grain (‘screenings’) for each plot was obtained as the weight of grain falling through a 2 mm-slotted screen after 40 shakes of a 80–100 g-sample on an industry-standard shaker. Grain protein concentration was estimated using Near-Infrared Spectroscopy (NIRS) and test weight calculated on the same harvest index grain. Plots were end-trimmed at maturity to *c*. 5.4 m in length and the outside border rows were removed before machine harvesting. Individual grain weight was determined from 200 random grains from the harvest sample and grain number m^–2^ was subsequently calculated from plot yields.

Immediately prior to harvest, 20 random heads were carefully harvested and stored from centre rows in plots in the MEF. These heads were air-dried at 35 °C before weighing and counts made of (i) numbers of fertile and infertile spikelets and (ii) numbers and weights of grain in first (a), second (b), and tertiary (c, d, and e) floret positions where present for two central and two distally located (upper) spikelets on each ear. Awn length was also measured for the two central spikelets. All ears were carefully hand-threshed using a rubbing-board and then aspirated to retain all grain. Ear harvest index (grain weight÷ear dry weight) was calculated for each sample.

Additional phenotyping was undertaken in Mexico of leaf chlorophyll content at booting using a Minolta® SPAD meter and leaf waxiness scored at flowering from 1 (no waxiness) to 5 (extreme waxiness).

### Glasshouse trial: measurement of spikelet size

In a separate study, an awned–awnletted NIL pair in the EGA Gregory commercial background was grown under favourable conditions in large 15L pots in a glasshouse (22/15 °C day/night). Five random heads were carefully harvested at developmental stages Z45, Z51, Z55, and Z60 (awnletted sib was harvested at Z60 only), and immediately dried in a fan-forced oven at 70 °C for 3 d. Central and distal (upper) spikelets were carefully removed from the rachis and up to six awns then separated from each spikelet before weighing and then awn lengths were measured with a ruler. Awn surface area was calculated assuming the shape of a triangular prism (i.e. 3×0.5×awn breadth×awn length) after Li *et al.* (2010*b*). Sides of the triangle were estimated at the base of each awn after averaging two measurements using a calibrated electronic micrometer. Glume and lemma lengths and widths were also measured on the same spikelets. The surface area of the glume and lemma was calculated after Teare *et al.* (1972) as a half ellipse each (i.e. surface area=(∏×(length×width))/2) to account for the exposed surface areas.

### Statistical analysis

Data were analysed statistically after first checking for normality and error variance heterogeneity across environments. The range in error variances was not large (less than 10; data not shown) and so data were left untransformed except percentage screenings which was arcsine transformed (for non-normally distributed percentage data) before analysis. A combined analysis of variance was then undertaken over environments for all agronomic and seed quality traits. Check cultivars and parents were eliminated from the analysis leaving only near-isogenic awned and awnletted pairs in four backgrounds. In the combined analysis, the presence (awned) or absence (awnletted) of awns was considered as a fixed effect, and crosses and environments were considered random effects. Expected mean squares were derived following Schultz (1955), and errors for statistical testing of specific main and interaction effects were ascertained from the expected mean squares. Following confirmation of significant among-entry differences, specific comparisons were made among NILs using pre-planned, single degree of freedom contrasts using the SAS procedure GLM (SAS Institute Inc., 1990). Contrasts were not orthogonal but emphasized meaningful partitioning into differences between mean progeny response and simple effects associated with either awned or awnletted NILs. Canopy temperatures were estimated for NILs after fitting time of assessment in the full linear model (after Rebetzke *et al*, 2013*b*). Where reported, genetic correlations and their standard errors were estimated (Holland, 2006). Unless otherwise stated statistical significance was at *P*=0.05.

## Results

A combined statistical analysis was undertaken for all genotypes (NIL pairs in all backgrounds) across the 25 sampled environments. These analyses and subsequent means are summarized for environments, partitioning of awn NILs, genetic backgrounds and their interaction, and interactions with environments, and the range in genotype means for the main environment groups. Coefficients of determination for the generalized linear models were commonly large (*R* ^2^=0.56–0.91) reflecting the large proportion of the total variation accounted for by treatments (genotypes, environments and their interaction).

### Among environment differences

**Fig. 1.**
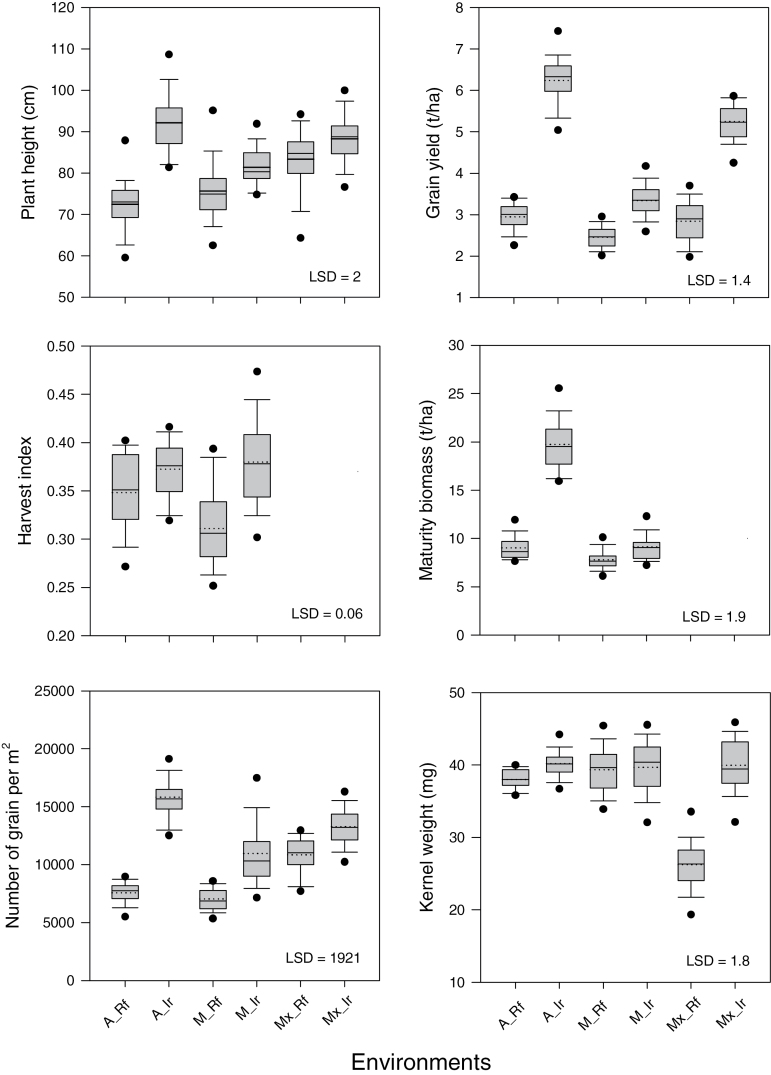
Boxplots summarizing the range in genotype means and environment means across Australian rainfed (‘A_Rf’) and irrigated (‘A_Ir’), Managed rainfed (‘M_Rf’) and irrigated (‘M_Ir’), and Mexican rainfed-drought (‘Mx_Rf’) and irrigated (‘Mx_Ir’) environments for growth and yield-based characteristics measured on near-isogenic wheat pairs varying for the presence and absence of awns, and representing three to four genetically-contrasting wheat backgrounds. Harvest index and maturity biomass were not measured in Mexico. LSD is the least significant difference for testing among genotype×environment means at *P*=0.05.

The range in environment means was large and statistically significant (Fig. 1; Table 1). Mean grain yield across all environments was 4.22 t ha^−1^ with a range of 1.38–7.93 t ha^−1^ (Fig. 1). The large range reflected differences in the timing and quantity of available water from rainfall and irrigation across sites and years and the different sets of NILs. For example, the irrigated Australian experiments containing up to 45 NIL pairs averaged grain yields of 6.29 t ha^−1^. In these environments, harvest index and total biomass averaged 0.385 and 16.3 t ha^−1^, respectively. Average grain number (15 807 grain m^−2^) and kernel size (39.9mg) (Fig. 1) were also high with supplemental irrigation as were numbers of spikes (341 m^−2^) and grain number per spike (46.4) (data not shown).

**Table 1.** Mean squares from analysis of variance for different agronomic traits measured on awned and awnletted near-isogenic lines (NILs) representing different genetic backgrounds and assessed in multiple environments

Source	Anthesis biomass^†^	Plant height^†^	Grain yield	Total biomass	Harvest index	Number of spikes^†^	Kernel weight	Number of grains^††^	Test weight	Grain screenings	Grains per spike	Canopy temperature
Environment (Env)	277**	13.66**	786**	4997**	0.123 ns	264 ns	5022**	3934**	214**	660**	74525**	186**
Experiment/Env	1802**	5.31*	70**	691**	0.181**	1178**	713**	479**	615**	345**	22789**	1713**
Near-isogenic lines	20 ns	1.0**	2.90 ns	6 ns	0.006 ns	88**	762**	29*	56**	90**	798**	8.6*
Awn	0.2 ns	0.04 ns	3.1 ns	0.5 ns	0.001 ns	3 ns	1144**	49*	20*	69*	1124*	5.7**
Background (Bgrd)	37.8 ns	2.25**	5.1 ns	7.4 ns	0.013#	201**	1057**	31 ns	76#	186*	1597*	3.8*
Awn×Bgrd	8.8 ns	0.03 ns	0.1 ns	6.5 ns	0.001 ns	3 ns	85**	4 ns	37#	3 ns	62 ns	0.2 ns
NILs×Env	24*	0.1**	2.34**	9 ns	0.004#	14**	54**	20**	23**	18**	236*	3.2*
Awn×Env	9.7 ns	0.01 ns	0.95 ns	5.2 ns	0.002 ns	6 ns	37**	10 ns	1 ns	5 ns	144 ns	3.1*
Bgrd×Env	49.3**	0.37**	3.76**	10.0 ns	0.004*	27**	89**	36**	32**	37**	447**	1.3 ns
Awn×Bgrd×Env	3.2 ns	0.04 ns	0.56 ns	9.2 ns	0.004 ns	4 ns	13 ns	4 ns	22*	3 ns	57 ns	1.1 ns
Pooled error	13	0.06	0.92	14	0.003	6	7	5	11	3	143	25
*R* ^2^	0.80	0.74	0.81	0.77	0.61	0.75	0.86	0.78	0.56	0.71	0.78	0.93

#, *, **: Mean squares statistically different from zero at *P*=0.10, 0.05, and 0.01, respectively; ns denotes not statistically different at *P*=0.05.

^†^: Mean squares×10^3^; ^††^ mean squares×10^6^.

Across the Managed Environment Facilities (MEFs), grain yield varied significantly from 1.38 t ha^–1^(Merredin 2012) to 4.49 t ha^−1^ (Narrabri 2012). On average, irrigated yields were 2.86 t ha^–1^and rainfed 2.54 t ha^−1^, representing a significant but small yield increase with supplemental irrigation of *c.* 14%. This greater yield was associated with significantly increased grain number (8 274 grains m^–2^ versus 7 949 grains m^−2^ for irrigated and rainfed, respectively) and grain size (40.1mg versus 37.7mg), and significant increases in both harvest index (0.36 versus 0.33) and total biomass (8.40 t ha^–1^ versus 8.01 t ha^−1^). Over all the MEF experiments, the greater yield of the irrigated trials reflected increases in both grain number (*r*_e_=0.39, *P* <0.01) and grain size (*r*_e_=0.53, *P* <0.01). Grain screenings varied from 0.49% (Yanco 2013) to 9.36% (Narrabri 2013), numbers of spikes from 173 m^–2^ (Merredin 2013) to 478 m^−2^ (Narrabri 2012), and grains per spike from 13.8 (Yanco 2012) to 34.0 (Merredin 2013). Across all environments and entries, the environmental correlations (*r*_e_) for percentage screenings was large and significant (*P* <0.01) with kernel size (–0.72), number of grains (0.89), and grain number per spike (0.97).

In Obregon (Mexico), grain yields of the irrigated and droughted experiments differed significantly averaging 5.25 t ha^–1^ and 2.89 t ha^−1^, respectively. The increased yield reflected a small but significant increase (+17%) in numbers of grain (13 265 grains m^–2^ versus 10 866 grains m^−2^) and a large increase (+34%) in average grain size (40.0mg versus 26.6mg).

### Between genotypes

The extent of genotypic variation across the assessed NILs varied depending on the trait under consideration (Tables 1, 2). For example, genotypic differences in plant height were largely associated with average differences between genetic backgrounds with Silverstar-derived NILs being taller on average (91cm) than Janz and Westonia NILs (81cm and 83cm, respectively; data not shown). Awned and awnletted NILs were approximately the same height when averaged across all environments (Table 2).

**Table 2.** Agronomic and grain quality means measured on up to 45 near-isogenic pairs varying for presence of awns and representing four contrasting recurrent parents evaluated across multiple environments in multiple years Values in parenthesis represent means for irrigated environments.

Parameter	Days to anthesis (d)	Plant height (cm)	Lodging score (1– 9)	Grain yield (t ha^–1^)	Total biomass (t ha^−1^)	Harvest index	No. of grains (no. m^–2^)	No. of spikes (no. m^−2^)	Grains per spike (no. spike^−1^)	Kernel weight (mg)	Grain screenings (%)	Test weight (kg.hectolitre-1)
**All environments**												
+Awns	131	81	3.2	3.85	12.6	0.354	10048	336	29.9	38.3	3.69	80.2
–Awns	132	82	3.3	3.81	12.7	0.355	10392	339	30.6	36.7	4.53	79.7
*t* test	ns	ns	ns	ns	ns	ns	*	ns	**	**	**	*
**Individual environment types**												
**(a)Australian rainfed/irrigated environments (*n*=45 NILs**)												
+Awns	129 (131)	72 (91)	2.3 (6.1)	2.84 (6.27)	7.90 (16.5)	0.362 (0.381)	7332 (15367)	304 (336)	24.1 (45.7)	39.0 (40.8)	4.90 (2.61)	80.8 (80.0)
–Awns	130 (131)	73 (92)	2.3 (6.1)	2.74 (6.32)	7.59 (16.2)	0.361 (0.391)	7385 (16247)	305 (345)	24.3 (47.1)	37.1 (38.9)	5.77 (3.22)	80.3 (79.8)
*t* test	ns (ns)	ns (ns)	ns (ns)	ns (ns)	ns (ns)	ns (ns)	ns (**)	ns (ns)	ns (*)	** (**)	** (**)	* (ns)
**(b) Australian Managed Environment Facilities (*n*=16 NILs**)												
+Awns	133 (133)	76 (77)	1.9 (2.8)	2.55 (2.91)	8.16 (8.45)	0.330 (0.349)	7847 (7982)	345 (328)	23.3 (25.9)	38.5 (40.9)	4.52 (3.07)	79.0 (80.9)
–Awns	133 (132)	76 (77)	1.8 (2.9)	2.52 (2.80)	7.87 (8.36)	0.336 (0.348)	8101 (8566)	344 (316)	24.3 (27.4)	36.8 (39.2)	5.75 (3.51)	78.6 (79.7)
*t* test	ns (ns)	ns (ns)	ns (ns)	ns (ns)	ns (ns)	ns (ns)	# (**)	ns (ns)	* (*)	** (**)	** (*)	* (**)
**(c) Mexico (Obregon) (*n*=36 NILs**)												
+Awns	84 (77)	83 (88)	–	2.95 (5.26)	–	–	10748 (12999)	–	–	27.4 (41.3)	–	–
–Awns	83 (77)	85 (89)	–	2.71 (5.23)	–	–	11244 (13533)	–	–	24.1 (38.7)	–	–
*t* test	ns (ns)	ns (ns)		* (ns)			# (**)			** (**)		

#, *, **: Awned and awnletted means are statistically different at *P*=0.10, 0.05, and 0.01, respectively; ns denotes means are not statistically different at *P*=0.05.

Subsequent assessment of genotypic variation and comparisons between awned and awnletted NILs will be reported ontogenetically (Table 3):

**Table 3.** Means for vegetative growth, anthesis biomass, and water-soluble stem carbohydrates (WSC) measured on 16 near-isogenic pairs varying for the presence and absence of awns, and representing four genetically-contrasting wheat backgrounds in droughted and irrigated (in parenthesis) environments in the Managed Environments Facilities (at Merredin, Narrabri and Yanco in 2012–13)

Awn phenotype	Early-vegetative		Mid-vegetative		Late-vegetative		Anthesis		
	Ground cover (%)	NDVI	Ground cover (%)	NDVI	Ground cover (%)	NDVI	Anthesis biomass (g m^–2^)	WSC concentration (%)	WSC content (g m^−2^)
+Awns	16 (17)	0.19 (0.19)	52 (50)	0.48 (0.50)	78 (83)	0.74 (0.73)	451 (590)	22.9 (24.2)	103 (143)
–Awns	18 (18)	0.18 (0.19)	54 (51)	0.50 (0.50)	79 (81)	0.74 (0.74)	449 (582)	23.3 (23.8)	105 (139)
*t* test	ns (ns)	ns (ns)	ns (ns)	ns (ns)	ns (ns)	ns (ns)	ns (ns)	ns (ns)	ns (ns)

ns: Awned and awnletted means are not statistically different at *P*=0.05.

#### (a) Vegetative growth

Measurements of vegetative growth were undertaken and reported for the MEF (Table 3). Growth from soon after seedling emergence to anthesis was the same for awned and awnletted NILs that had similar anthesis biomass (Tables 1, 3) and tiller numbers (data not shown). These similar anthesis biomass estimates were consistent across rainfed and irrigated environments and were similar across the four genetic backgrounds sampled (Table 1; data not shown). In Obregon, Mexico, similar leaf chlorophyll contents (45.6) were observed for awned and awnletted NILs while awnletted NILs produced small but significantly greater leaf waxiness scores under both rainfed (cf. 2.82 and 3.11 for awned and awnletted NILs, respectively) and irrigated (cf. 3.50 and 3.72) conditions (data not shown).

Averaged across all environments, the estimates of stem water-soluble carbohydrate (WSC) concentration at anthesis were 23.53% and 23.58% for awned and awnletted NILs, respectively. The WSC concentration was significantly greater in the Frame and Westonia backgrounds (25.40% and 25.51%, respectively), and smallest for the Janz and Silverstar NILs (21.01% and 21.80%, respectively). The similar WSC concentration and anthesis biomass for awned and awnletted NILs translated to statistically similar stem carbohydrate contents for awned and awnletted NILs alike (Table 3).

#### (b) Canopy temperature

Differences between environments and sample dates for mean canopy temperature were large (12.1–32.5 °C) and highly significant (*P* <0.01). The environments in Obregon were particularly warm (canopy temperatures of 22–33 °C) whereas canopy temperatures in Australia varied from 12–28 °C and included a single (21.00–22.00h) night canopy temperature of 13.0 °C (Fig. 3). In turn, significant genotypic differences were observed for canopy temperature across environments (Table 1; Fig. 3). The entry (genetic) variance (± se) was 0.065±0.018, and broad-sense heritability on an entry-mean basis was 0.64±0.14 (*P* <0.01). A large component of the genetic variance for CT was associated with genetic background (Table 1) with Janz NILs averaging a significantly cooler CT of 23.57 °C compared with the warmer CT of Frame (23.73 °C) and Westonia (23.78 °C) NILs. Over the broad range of environments sampled, mean CT was significantly cooler for awned (23.54 °C) than awnletted (23.81 °C) NILs and these differences were consistent across backgrounds (Table 1). Despite the average difference in CT in awned NILs, the robustness of these differences were strongly dependent on prevailing air temperature with awnletted wheats producing as cool or cooler CT than awned wheats with cooler ambient canopy temperatures (Fig. 3). Indeed, when regressed against the mean CT of all NILs for each of the 25 sampled environments, the value of awns in canopy cooling increased linearly (*r*_p_=–0.55, *P* <0.01) with increasing air temperatures. The single CT observation undertaken on a cooler, calm, and cloudless night indicated awnletted NILs to be significantly cooler than for awned wheats (Fig. 3).

#### (c) Maturity biomass and grain yield

**Fig. 2.**
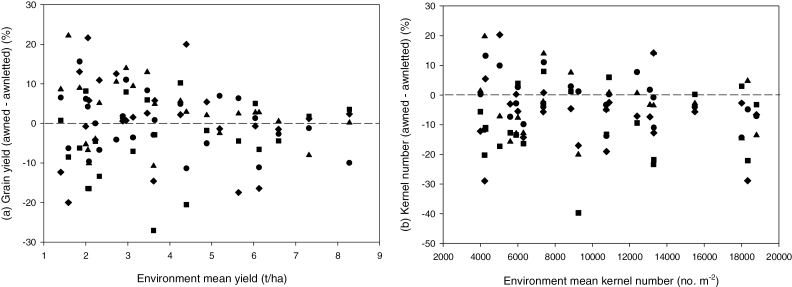
Awned–awnletted NIL mean differences (expressed as % of awned NILs) for grain yield and kernel number across up to 23 environment means for the four different genetic backgrounds [Frame (solid circles), Janz (solid triangles), Silverstar (solid squares) and Westonia (solid diamonds)]. Negative values indicate awned wheats are reduced relative to awnletted sibs.

Across all environments, awned and awnletted NILs were not statistically different for grain yield, harvest index, and total maturity biomass (Table 1). There was a significant reduction (–8%) in grain yield for awnletted wheats under irrigation in Mexico (Table 2). Figure 2 summarizes grain yield differences for awned and awnletted NILs across all environments. There was no obvious yield benefit with yield level of the individual environment. Genetic background effects were commonly small and non-significant although Frame produced a marginally, albeit significantly, smaller harvest index (0.341) than the other three backgrounds which ranged from 0.354 (Westonia) to 0.364 (Janz). Despite the equivalent grain yields, differences were large and statistically significant among entries for kernel number and size (Table 1). Across all environments, awned wheats produced significantly larger but fewer kernels than awnletted sibs (Table 2). The difference in grain number was more pronounced in higher-yielding, irrigated environments while the larger grain size of awned sibs was consistent across all environments and irrigation regimes. For example, in side-by-side drought-irrigated experiments in Mexico and the MEF, kernel weight advantage in awned wheats was always maintained at 5–10% greater than for awnletted NILs (Table 2). Kernel weight was significantly greater in the Westonia background and smallest in the Silverstar background (data not shown) while the influence of awns was statistically greater in the Silverstar background (+4.1%) and smallest in the Frame background (+0.3%) (data not shown). Across all environments and entries, kernel weight and number were negatively correlated (*r*_g_=–0.55, *P* <0.01). The genetic correlation for kernel weight and grain yield was small (*r*_g_=0.13, *P* <0.05) while the genetic correlation for grain yield and kernel number was moderate (*r*_g_=0.61, *P* <0.01).

Numbers of fertile spikes were the same for awned and awnletted NILs (Tables 1, 2). Much of the observed genetic variation was due to background differences, in particular, the contrast of Westonia NILs (291 spikes m^−2^) with those of Janz and Frame (350 and 359 spikes m^−2^, respectively). Hence, increased grain number was due to significantly more kernels per spike for awnletted NILs (Tables 1, 2).

Genotypic variation was large for grain screenings (Table 1). Awnletted wheats produced significantly greater screenings and this was common for all environments both irrigated and droughted (Table 2). Across backgrounds, Silverstar produced significantly greater screenings (5.6%) whereas Frame had the smallest percentage (3.1%) (data not shown). Despite this difference, the influence of awns on the reduction in screenings was consistent across all backgrounds (Table 1). The background×environment interaction was significant largely because of the relatively greater screenings of Silverstar NILs under drought (data not shown). Across all environments and NILs, grain screenings and kernel weight were negatively genetically correlated (*r*_g_=–0.53, *P* <0.01) while kernel number and grain screenings were also strongly correlated (*r*_g_=0.93, *P* <0.01). Similarly, genotypic increases in grain number per spike and grain screenings were strongly correlated (*r*_g_=0.91, *P* <0.01).

Test weight was marginally, albeit significantly, smaller in awnletted NILs (Table 1). This difference was *c.* 1% in size (Table 2) with test weight being greatest in the Frame (80.6) and smallest in the Janz (79.6) genetic backgrounds. The awned phenotyped was associated with significantly greater test weight in all genetic backgrounds (+0.36– 1.17%) except in the Janz background where awned NILs were significantly smaller (–0.5%) for test weight. Grain protein concentration was significantly greater in awned (13.39%) than in awnletted (13.15%) NILs and this difference was consistent in irrigated and rainfed environments and across all genetic backgrounds (data not shown). The influence of awns on grain protein concentration varied across backgrounds and was strongest in the Frame and Westonia backgrounds (data not shown). Across all entries and environments, increases in screenings were associated with genotypic reductions in test weight (*r*_g_=–0.58, *P* <0.01).

#### (d) Ear morphology

The influence of irrigation on most ear characteristics was not large (Table 4) and approximately consistent in size (10–15%) reflecting the broader differences observed for grain yield and grain yield components in the larger NIL sets (Table 2; Fig. 2). Consistent with these observations was the small and commonly statistically non-significant interaction of irrigation with awn type for ear morphology (Table 4).

**Table 4.** Awned and awnletted means for spike and spikelet characteristics measured at maturity on 16 awned-awnletted NILs representing four genetic backgrounds from rainfed and irrigated (in parenthesis) environments in the three Managed Environment Facilities (at Merredin, Narrabri and Yanco) in 2012–2013

	Awn length (mm)	Total number of spikelets (*n*)	Number of fertile spikelets (*n*)	Number of sterile spikelets (*n*)	Central spikelets (per spikelet)			Distal spikelets (per spikelet)			Ear harvest index
					Total grain weight (mg)	Number of grains (*n*)	Average grain weight (mg)	Total grain weight (mg)	Number of grains (*n*)	Average grain weight (mg)	
					wt						
+Awns	59 (64)	18.9 (19.3)	14.9 (16.2)	3.94 (3.11)	110 (122)	2.98 (3.14)	37 (39)	50 (61)	1.53 (1.91)	33 (32)	0.672 (0.694)
–Awns	5 (7)	18.6 (19.1)	15.7 (16.7)	2.90 (2.31)	106 (122)	3.19 (3.40)	33 (36)	50 (63)	1.78 (2.16)	28 (29)	0.723 (0.744)
*t* test	** (**)	ns (ns)	* (#)	** (**)	ns (ns)	# (*)	** (*)	ns (ns)	* (**)	** (*)	** (**)

#, *, **: Means are statistically different at *P*=0.10, 0.05, and 0.01, respectively; ns denotes means are not statistically different at *P*=0.05.

Average awn length was significantly greater for awned NILs (56mm versus 2mm), and, on average, was significantly greater in length for Westonia (38mm) and shorter for the Silverstar (29mm) genetic backgrounds. Frame and Janz were intermediate in awn length (33mm) (data not shown). Total numbers of spikelets were not different for awned and awnletted sibs but the numbers of fertile spikelets (i.e. spikelet with at least one grain) was significantly (*P* <0.01) greater for awnletted lines (86.1% versus 81.4% for awnletted versus awned NILs, respectively) (Table 4). Any sterile spikelets were basal (data not shown). The greater number and higher percentage of fertile spikelets for the awnletted sibs was consistent across all genetic backgrounds (data not shown).

A detailed examination of individual spikelets indicated that awned and awnletted sibs produced approximately the same total grain weight in both central and distal spikelets and in both irrigated and rainfed treatments (Table 4). However, numbers of grains were significantly greater for awnletted lines (+7.1% and +12.8% for central and distal spikelets, respectively) which was largely compensated for by a reduction in average grain size (–9.8% and –14.1%, respectively). Closer examination of the central spikelets highlighted that awnletted sibs tended more often to produce grains at terminal floret position 4 (59% versus 46% for awnletted and awned NILs; data not shown). In distal spikelets, awnletted wheats more often produced grains at floret 2 (97% versus 83%), and floret 3 (22% versus 14%). Awnletted wheats occasionally produced a grain at the 4th and 5th florets of the central (2%) and distal (1%) spikelets whereas no grain was produced at these positions in the awned NILs. Total weight per ear was not different for awn and awnletted NILs (data not shown) but threshed grain weight per ear was significantly greater for awnletted (21.9g) than awned (19.7g) NILs. The increased grain yield per ear for awnletted NILs translated to a significantly greater ear harvest index for these wheats (Table 4). In all cases, there was no significant awn NIL×background interaction (data not shown).

Between three and four awns were produced on the central and distal spikelets of awned and awnletted NILs (data not shown). Awn lengths for central spikelets was 56mm and 2mm, on average, for all awns on the awned and awnletted NILs at Z60. Average awn lengths on distal spikelets were similar to the central spikelet for awned NILs (52mm) but were longer for awnletted wheats (9mm). Awns were greatest in length at development stage Z60 and the longest awns of extreme NILs measured 73mm and 69mm for the central and distal spikelets, respectively (data not shown).

The dynamics of spikelet biomass and surface area were analysed in more detail in an awned–awnletted EGA Gregory NIL pair grown under favourable glasshouse conditions (Table 5). Spikelets increased in size, almost doubling in dry weight between the spike development stage (Z45) to the commencement of anthesis (Z60) (Table 5). This relative increase in spikelet biomass was similar for both central and distal spikelets alike. The proportion of total spikelet dry weight that was awn tissue was greatest earlier in spike growth (*c.* 42% at Z45) and decreased linearly to approximately 30% by Z60, and was similar for central and distal spikelets. The surface area of spikelet awns increased linearly from Z45 onwards (Table 5) reflecting small increases in awn length and diameter (data not shown). Similarly, glume and lemma size increased up to Z60 to increase the total surface area of the spikelet (excluding awns) (Table 5). The surface area of awns was proportionally smaller in central than for distal spikelets. By Z60 the surface area of the central spikelet was *c.* double the size of the distal spikelets. Spikelets from the pair of EGA Gregory NILs had similar dry weights and surface areas for the non-awned components of the spikelet (Table 5). However, as expected, the awns had considerably greater dry weight and surface area in the awned NIL.

**Table 5.** Dry weight (mg) and photosynthetic surface area (mm^2^, in parenthesis) for spikelets (excluding awns, i.e. glume, lemma, and palea) and awns measured on central and distal spikelets assessed from Z45–Z60 for an awned EGA Gregory NIL (Z45–Z60) and its awnletted sib (Z60 only) Data from a glasshouse experiment.

Spikelet	Organ	Awned NIL			Awned or awnletted NIL	
		Z45	Z51	Z55	Z60 (+awn)	Z60 (–awn)
Central	Glume+Lemma^*a*^	13.8 (42.2)	20.7 (47.9)	25.0 (53.7)	31.2 (61.4)	21.8 (50.3)
	Awn	9.6 (34.1)	10.2 (37.2)	12.1 (42.7)	12.9 (43.3)	2.7 (11.4)
	% Awn	41.0 (43.9)	33.1 (43.1)	32.6 (44.2)	29.2 (41.3)	11.0 (16.7)
Distal	Glume+Lemma^*a*^	9.2 (21.2)	11.0 (23.1)	13.9 (24.3)	15.8 (26.8)	10.9 (26.7)
	Awn	6.7 (27.3)	6.8 (28.2)	6.7 (34.8)	6.7 (35.4)	1.3 (6.4)
	% Awn	42.1 (55.2)	38.2 (54.9)	32.5 (57.7)	29.7 (57.2)	10.6 (18.8)
	*t* test	ns (*)	* (*)	ns (*)	ns (*)	ns (ns)

*: Means are statistically different at *P*=0.05; ns denotes means are not statistically different at *P*=0.05.

^*a*^ Dry weights included glume, lemma, and palea whereas photosynthetic area was based on glume and lemma only.

## Discussion

### The role of awns as photosynthetic tissue

Awns are filiform, bristle-like extensions of the lemma found in many winter grasses and cereals. Genetic control of awn length in bread wheat is relatively simple with awn suppression under the control of dominant alleles at loci on chromosomes 4AS (*Hd*, hooded), and chromosomes 5AL and 6BS (*B1* and *B2*, tipped 1 and 2, respectively) (Sourdille *et al.*, 2002). Background genetic effects are also important in the lengths of individual awns (Blum, 1985) as shown here with the contrasting awn lengths of Westonia and Silverstar NILs. Indeed, the awn lengths reported here are consistent with the range in awn lengths reported for bread wheat genotypes elsewhere (Ali *et al.*, 2010; Maydup *et al.*, 2014).

Owing to their triangular shape awns can provide a potentially large photosynthetic surface comprising up to 60% of the total ear surface area (Blum, 1985; Motzo and Giunta, 2002), and their location above the canopy where light and CO_2_ are non-limiting permits the potential for maximal assimilation. Their contribution has been reported to be as much as 60% of total spike photosynthesis (Teare *et al.*, 1972), and up to 12% of total canopy photosynthesis of field-grown wheat plants (Evans *et al.*, 1972; Olugbemi *et al.*, 1976). However, Teare *et al.* (1972) reported that increases in spike photosynthesis of awned wheat NILs was reasonably compensated by a near-doubling in transpiration (reflecting the larger transpiring area) to reduce the WUE of awned spikes. In terminal drought conditions, greater photosynthesis in awned barleys was associated with significantly reduced ear dry weight at maturity (Hosseini *et al.*, 2012). However, this contrasts with reported benefits of awns for increasing photosynthesis and grain yield for barleys grown under irrigated conditions (Bort *et al.*, 1994).

### Influence of awns on grain yield and components

Many of the studies reporting comparisons of awned and awnletted wheats were undertaken using unrelated varieties or one or few NILs from a single genetic background. Further, studies were commonly undertaken in spaced plants in the glasshouse and rarely in plots across a large range of potential grain yield conditions. In our study, we assessed performance of up to 45 awned and awnletted NILs generated in four diverse genetic backgrounds and evaluated it across 23 contrasting rainfed and irrigated environments. Despite the large genetic diversity and the range in grain yield from 1.38 t ha^–1^ to 7.93 t ha^−1^, there was little difference in average yield of awned and awnletted wheat NILs. There was an indication of improved performance for awned wheats in some environments (e.g. warmer, drier, rainfed environments in Obregon, Mexico) while awnletted NILs appeared to be better adapted to higher-yielding, favourable environments. However, overall, yield differences between awned and awnletted NILs were commonly small, reflecting similar leaf area and tiller development, anthesis and maturity biomass, and harvest index among NILs.

Despite the similarities in yield, awnletted wheats produced significantly more grains but of smaller average size. In turn, more grains were produced per spike reflecting the production of significantly more fertile spikelets coupled with a greater frequency of grains in tertiary (c, d, and e) florets. This increased grain number was associated with grains of a smaller size particularly those located in these tertiary florets. The association for grain size and awns was not consistent across genetic backgrounds with the difference in grain size being greatest in the Silverstar and smallest in the Frame backgrounds. The smaller grains in awnletted NILs and particularly those in the small-grained genetic background Silverstar contributed to a significantly higher proportion of screenings in these genotypes. Silverstar was a prominent Australian wheat variety notoriously poor for the production of high screenings (Mitchell *et al.*, 2013). Screenings arise from small or shrivelled grains and are commonly produced in more distal florets (Sharma and Anderson, 2004; Mitchell *et al.*, 2012). They tend to be more common under conditions of environmental stress, particularly drought (Sharma and Anderson, 2004). Stem carbohydrates can contribute to reduce the frequency of screenings (Rebetzke *et al.*, 2008) but stem carbohydrates were not different between awned and awnletted NILs.

Grain size and screenings were genetically correlated (*r*_g_=–0.47, *P* <0.01) across all NILs. This is consistent with earlier reports (Mitchell *et al.*, 2012; 2013) of strong negative genotypic correlations for grain size and screenings. Awned wheats are commonly reported to produce larger grain weights, as shown for multiple backgrounds (Knott, 1986; Olugbemi and Bush, 1987; Martin *et al.*, 1993;Weyhrich *et al.*, 1994; 1995; Motzo and Giunta, 2002; ) but this is often at the expense of fewer grains (Teich, 1982; Martin *et al.*, 1993; Weyhrich *et al.*, 1994; 1995). Here, reduced grain size in awnletted wheats was associated with a significant reduction in grain diameter (Martin *et al.*, 1993), an important determinant of grain screenings (Sharma and Anderson, 2004; Mitchell *et al.*, 2012). In addition, increased grain number reflected increased grain number per spike (Weyhrich *et al.*, 1995). Overall, the larger grain numbers produced by all awnletted NILs was associated with reduced grain filling leading to smaller grains, a trade-off observed in many studies (Slafer *et al*., 2014). Further, we observed that the smaller grains in the awnletted NILs were commonly those grains that glumes adhered to and, presumably, were in distal floret positions. Reduced threshability has sometimes been reported anecdotally as a problem with awnless wheat breeding lines.

### Role of awns in spike biomass partitioning

Spike chaff consists of awns, glumes, palea and lemma, rachis and rachillas, and can contribute between 6% and 54% of total spike weight at maturity (McMaster, 1997). Wheat awns can be very large in size (e.g. up to 13cm in length in some durum wheats; Motzo and Giunta, 2002). They commence elongation soon after floral initiation (McMaster, 1997) and their final sizes are determined largely in advance of their appearance and the commencement of photosynthesis at development stage Z50. Because awns are proportionally very large early in spike development (up to 40% of total spikelet biomass of central and distal spikelets; Table 5) they can represent a significant sink competing with growing florets for the available assimilates.

In awned NILs, the reduced grain number per spike reflected fewer fertile (basal) spikelets and fewer, fertile tertiary florets. The basis for this reduced fertility is unclear. The allocation of assimilate between competing structures through reproductive growth has long been debated. Floret death contributing to reduced fertility has been hypothesized by Kirby (1988) and others (Fischer and Stockman, 1986; Miralles *et al.*, 1998) to be sensitive to competition for assimilate between elongating stems and growing florets, and confirmed with shading studies (Gonzalez *et al.*, 2011). Arisnabarreta and Miralles (2006) reported the importance of larger ovary size in survival and subsequent grain-setting in distal spikelet positions of barley ears. Given the globally near-ubiquitous increase in wheat yields through indirect selection for grain number, future yield gains should be achievable through increases in the potential rate of floret survival (Sreenivasulu and Schnurbusch, 2012). Even within the growing ear, movement of carbon to a large sink such as a growing awn could compromise floret growth and survival particularly in tertiary florets (and beyond). Indeed, comparisons of barley awned NILs demonstrated that increasing awn length was associated linearly with increased kernel size but fewer kernels per spike consistent with a hypothesis for competition for assimilate between awn and floret development (Schaller and Qualset, 1975).

Given previous observations of greater photosynthesis in awned wheats (Evans, 1972; Blum, 1985; Maydup *et al.*, 2010) it could be expected that ear dry weights and harvest index would be greater for awned NILs. However, by maturity, ear dry weights were not different for awned and awnletted NILs but partitioning of total ear dry matter to grain (spike harvest index) was significantly greater for awnletted NILs (cf. 67% and 72% for awned and awnletted NILs, respectively)(Table 4). This greater ear harvest index in both irrigated and rainfed environments appeared to reflect a reduction in carbon allocation to awns in these NILs. This observation is not unique with Weyhrich *et al.* (1995) also reporting a greater spike harvest index for awnletted NILs in both well-watered (+3–5%) and water-stressed (+3–10%) regimes.

### Impact of awns on grain quality

Grain quality is a key consideration in the commercial release of a milling-based wheat variety. As indicated, awned NILs were consistently larger in grain size and this contributed to significant reductions in smaller, shrivelled grain or ‘screenings’. Test (or hectolitre) weight and grain protein concentrations were significantly (albeit marginally) smaller for awnletted NILs although the extent differed across genetic backgrounds. In other studies (Patterson *et al.*, 1962; Martin *et al.*, 1993; Weyhrich *et al.*, 1994), awned lines were greater for test weight and this was consistent across multiple genetic backgrounds. Awns have been associated with reductions in grain protein concentration (Knott, 1986; Weyhrich *et al.*, 1994) but this may simply reflect a larger dilution of nitrogen resulting from the greater yields observed in these studies. Flour yields, dough extensibility (Martin *et al.*, 1993), and flour sedimentation were not studied here but Knott (1986) found them to be similar for awned and awnless NILs. In an awned×awnless DH population, Ma *et al.* (2007) reported that the presence of awns at the 5A awned locus was associated with increased grain protein concentration and water absorption across multiple sites. Overall, the presence of awns had a positive effect on the grain commercial value with reduced screenings and increased test weight, while the effects on protein contents varied depending on the environments tested.

### The impact from awns on canopy temperature

The increased transpiration commonly reported for awned NILs (Blum, 1985; Weyhrich *et al.*, 1995) should translate into cooler spike temperatures. However, reliable measures of spike temperatures are difficult to obtain (Panozzo *et al.*, 1999) and so inference to spike temperature is usually made from canopy temperature (CT). Indeed, comparisons of awned–awnless NILs in barley (Ferguson *et al.*, 1973) and durum wheat (Motzo and Giunta, 2002) have demonstrated awns to be associated with a reduction in CT. We too observed awned NILs to be cooler with a small but significant average reduction in canopy temperature (23.54 °C and 23.81 °C for awned and awnletted NILs, respectively) across all environments. While this reduction was repeatable for all genetic backgrounds, it was mainly observed in environments where CT was relatively high (>25 °C; Fig. 2). The reduction in CT here was consistent with the reduction for one barley awned–awnless NIL (Ferguson *et al.*, 1973) but much smaller than for another barley NIL, and smaller than the average 0.9 °C reduction reported in long-awned, durum wheat NILs (Motzo and Giunta, 2002). Similarly, the small difference tha twe observed was consistent with comparisons of awned and mechanically de-awned spikes in two Argentinean wheat varieties (Maydup *et al.*, 2014). A greater awn length and therefore surface area, coupled with the warmer air temperatures (CT of 28–35 °C) may have contributed to the larger differential observed in the durum NIL study. In the two warmest environments sampled here (CT above 30 °C at Gatton ‘irrigated’ and Obregon ‘droughted’), the reduction in CT in awned NILs was modest at *c*. -0.5 °C.

**Fig. 3.**
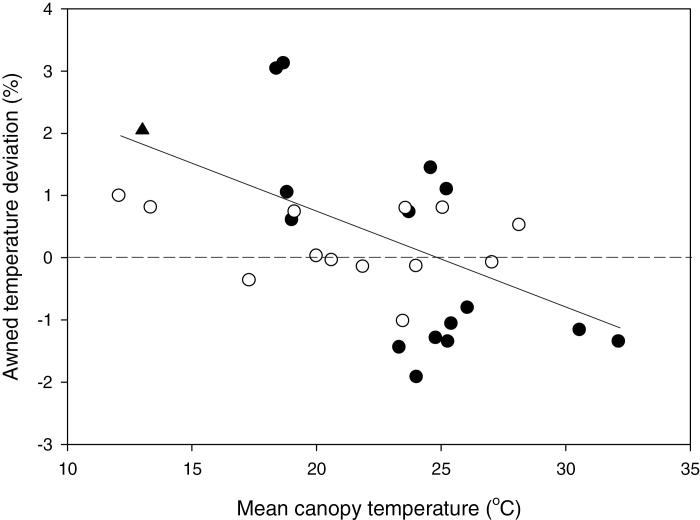
Awned–awnletted NIL canopy temperature (CT) deviation (expressed as a percentage of awned NIL CT) for day (open circles) and night (open triangles) sampling measured across the different experiments. Statistically different CT between awned and awnletted NILs (*P* <0.05) are denoted as filled symbols. The relationship for percentage temperature deviation and mean CT is: *y*=3.49–0.149*x* (*r* ^2^=0.33, *P* <0.01).

Awns provide a surface for cooling via greater transpiration. Ferguson *et al.* (1973) also hypothesized that some of the benefit of awns in spike/canopy cooling was in sensible heat transfer and the impact of awns on an altered boundary layer to affect air turbulence about the canopy. In our study, NILs were of comparable height and biomass, removing potential confounding effects of water use and boundary layer (Rebetzke *et al.*, 2013b). We observed that awnless NILs were reliably cooler at cooler air temperatures and that any significant cooling with awns only occurred at warmer canopy temperatures of above 23 °C (Fig. 3). Interestingly, Panozzo *et al.* (1999) reported spike temperature of an awned wheat variety was not different or was even warmer than an awnless variety in the same experiment at air temperatures below 40 °C. It is unclear if this contradiction reflects water stress or large genetic background effects in their study.

### Implications of selection of awnletted wheats with improved performance

A number of studies have highlighted the potential of awns to improve photosynthetic capacity and WUE to increase wheat performance. However, the adoption of awns in commercial breeding programmes has largely been restricted to regions that commonly encounter drought and high temperature stresses. That is, awned wheat varieties tend to be more common, although not exclusive, to drier, rainfed environments such as those of Australia, South America, and the USA, and the irrigated wheat programme at CIMMYT, and are less prevalent in wetter more humid environments particularly those encountered in northern Europe. The presence of awns have been linked to a predisposition to an increase in pre-harvest sprouting (King and Richards, 1984), greater disease susceptibility (Mesterhazy, 1995), and sensitivity to frost damage (Whaley *et al.*, 2004). Further, awns significantly reduce the capacity for grazing or cutting for hay of failed wheat crops damaged by drought, heat or frost. As a result, there is a preference by growers for awnless or awnletted wheat varieties in regions where crops are grazed or cut for hay/silage (Martin *et al.*, 1993; Weyhrich *et al.*, 1994).

The major benefit of awns was in the maintenance of a larger grain size to reduce screenings and thereby to increase harvestable or economic yield. Awns were also important in maintaining greater test weights and grain protein concentrations although the benefits here were not particularly large and are not always found in the literature (Knott, 1986; Weyhrich *et al.*, 1994). Here, awnless NILs that combined high yield potential (and grain number), reduced grain screenings, and high test weight and grain protein concentration were identified in all but the Silverstar background. Reduced glume adherence and increased threshability of awnless wheats may reflect mechanical changes in the structure of a spikelet or may simply reflect the smaller kernel size and diameter characteristic of awnless wheats. Other studies (Weyhrich *et al.*, 1994) identified a high proportion of awnless or awnletted NILs that were not statistically different from awned sibs for grain yield and quality.

The potential for awnless and awnletted wheats to increase floret survival and to increase grain number provides further opportunity for increasing grain yields in both low- and high-yielding environments alike. However, the opportunity and ease in identifying higher-yielding, commercially-ready awnless or awnletted wheats may be background-dependent necessitating larger population sizes and greater levels of inbreeding before marker-assisted selection and/or phenotypic selection. Further traits including intrinsically larger carpel size and reduced-tillering can be incorporated with the awnless phenotype to increase kernel size and to reduce screenings (Hendriks *et al.*, 2016; Mitchell *et al*., 2012; 2013).

## Conclusion

We have demonstrated that the awned phenotype contributes to slightly greater economic or harvestable yield through larger kernels and reduced screenings and, to a lesser extent, improved grain quality. However, opportunity exists in the selection of awnless wheats with comparable yield and quality, particularly in more favourable environments characteristic of northern Europe and in other regions where crops are irrigated. Such phenotypes have the benefit of being suitable for grazing, while their potential for reduced frost and disease susceptibility, and pre-harvest sprouting, may be of benefit in future changing climates.

## Abbreviations:

NILs: Near-Isogenic Lines
*r*_e_: environmental correlation
*r*_g_: genetic correlation
*r*_p_: phenotypic correlation
*R*^2^: coefficient of determination.
